# 

**Published:** 1841-12

**Authors:** 


### CONTENTS

### OF NO. VI.

---

### ORIGINAL COMMUNICATIONS.

### ESSAYS AND CASES.

ART. I.—Observations on the epidemic Yellow Fever of Natchez, and of the South-west. By JOHN W. MONETTE, M. D. &c., of Washington, Mississippi. - - 411

ART. II.—Fungus Hæmatodes removed by excision, and a radical cure effected. By JOHN TRAVIS, M. D., of Carroll County, Tennessee. - - - - - - 440

ART. III.—Insanity and Asylums for the Insane—Five Lectures. By W. A. F. BROWNE, Surgeon and Superintendent of the Montrose Asylum: Edinburgh, 1837. 443

### ORIGINAL INTELLIGENCE.

Alleged discharge of hairs from the hand. - - - - - - 483

The shower of flesh and blood. - - - - - - 485

Louisville Medical Institute. - - - - - - - - 487

New work on the climate of the United States, and its endemic diseases. - - - - - - - - - - 487

Spinal counter stimulation in Congestive Fever. - - - 487

Proposed enlargement of the Journal. - - - - - 488

To Corespondents. - - - - - - - - - - 488

